# Measurement of cystatin C functional activity in the cerebrospinal fluid of amyotrophic lateral sclerosis and control subjects

**DOI:** 10.1186/2045-8118-10-15

**Published:** 2013-03-15

**Authors:** Meghan E Wilson, Imene Boumaza, Robert Bowser

**Affiliations:** 1 Department of Pathology, University of Pittsburgh School of Medicine, Pittsburgh, PA, USA; 2 Division of Neurology, Barrow Neurological Institute, Phoenix, AZ, USA

**Keywords:** Cystatin C, Functional activity, Cerebrospinal fluid, Amyotrophic lateral sclerosis

## Abstract

**Background:**

Cystatin C is a constitutively expressed and abundant cysteine protease inhibitor within the cerebrospinal fluid (CSF). Recent studies have reported a significant reduction in cystatin C concentration in the CSF of patients with amyotrophic lateral sclerosis (ALS) and several other neurodegenerative diseases, relative to healthy controls. Cystatin C can exhibit both neuroprotective and neurotoxic properties, suggesting that altered CSF cystatin C concentrations could potentially impact the pathogenesis or progression of these disorders. However, it is unclear if alterations in cystatin C concentration result in physiologically relevant differences in its functional activity within the CSF. Measurements of the cysteine protease inhibitory activity of cystatin C within the CSF have not been reported, and the relationship between CSF cystatin C concentration and activity levels in different disease contexts has not been investigated.

**Methods:**

We used a papain inhibition assay to evaluate the total cystatin C activity in CSF samples from 23 ALS patients, 23 healthy controls, and 23 neurological disease controls. Cystatin C concentrations in these samples were previously measured by ELISA. Correlations between cystatin C concentration and activity were assessed with nonparametric statistics. Activity ratios were compared among diagnostic groups using both one-way ANOVA and repeated measures statistics.

**Results:**

Total cystatin C activity was found to be directly proportional to its protein concentration in all subjects, and cystatin C activity was not altered in ALS patients. In addition, our data suggest that cystatin C is the predominant cysteine protease inhibitor in human CSF.

**Conclusions:**

Our data demonstrate the successful measurement of the functional activity of cystatin C in the CSF, and show that total cystatin C activity can be inferred from its total protein concentration. Our results also suggest that cystatin C is the major cysteine protease inhibitor in human CSF and altered CSF cystatin C concentration may play a role in the pathobiology of ALS and other neurological diseases.

## Background

Cystatin C is a low molecular weight cysteine protease inhibitor that modulates protein degradation and cell-matrix interactions [1]. This protein binds with high affinity to papain, cathepsins B, H and L, calpain, and dipeptidyl peptidase, regulating both intracellular and extracellular cysteine proteases [2]. Cystatin C is produced by nearly all cells and is secreted into the extracellular space, blood, or cerebrospinal fluid (CSF). In the central nervous system (CNS), cystatin C is synthesized by choroid plexus cells and secreted into the CSF. Interestingly, cystatin C is 5-fold more concentrated in the CSF than in the bloodstream [3]. This suggests that cystatin C may have important functions within the CSF relating to the modulation of protein degradation and, potentially, the regulation of multiple signaling pathways [4].

We and others have reported that cystatin C levels are reduced in the CSF of patients with amyotrophic lateral sclerosis (ALS) when compared to both healthy controls and neurological disease controls [5-9]. Altered levels of CSF cystatin C have also been reported for other neurological disorders including multiple sclerosis, Alzheimer’s disease, and Creutzfeldt-Jakob disease [10-12]. The reduction of CSF cystatin C levels in patients with multiple sclerosis and other inflammatory diseases suggests that cystatin C has a role in modulating neuroinflammation. Indeed, some activity of this type has been demonstrated in cell culture models [13,14]. In addition, cystatin C has been shown to exhibit both neuroprotective and neurotoxic properties within *in vitro* and *in vivo* models. Therefore, cystatin C may have multiple functions relevant to the pathogenesis and progression of neurological disease. However, the influence of altered cystatin C levels in this context remains unclear.

While the alteration of CSF cystatin C concentration in various neurological disorders has been established, the relationship between the concentration and functional activity of this protein within the CSF is unknown. It is possible that CSF cystatin C exhibits simple, linear activity kinetics at all physiologic concentrations, such that it’s mean activity is a constant multiple of its total concentration. Alternatively, cystatin C may demonstrate concentration-dependent activity kinetics *in vivo*, resulting in activity differences that are either magnified or diminished relative to concentration differences. Along different lines, it is also possible that the post-translational modification or proteolytic processing of cystatin C is altered in some disease states, potentially resulting in altered baseline activity kinetics for the protein.

Once the concentration-to-activity relationship is clarified, it will be possible to draw conclusions about the potential effects of altered cystatin C concentration in neurological disease states. If the observed differences in CSF cystatin C concentration do result in significant changes in its cysteine protease inhibitory activity, this could directly impact the protein milieu of the CSF, alter the extracellular matrix within the CNS, and influence downstream signaling events, potentially generating either neurotoxic or neuroprotective effects.

To determine the relationship between cystatin C concentration and functional activity within the CSF, we modified a papain inhibition assay to enable the measurement of the protease inhibitory activity of cystatin C in this biofluid. We used this assay to evaluate cystatin C activity in CSF samples from a total of 69 ALS patients, healthy controls, and disease controls. We found that the functional activity of cystatin C within the CSF is directly related to its protein concentration, and that reduced cystatin C protein levels in the CSF of ALS patients likely correspond to reduced cysteine protease inhibitory activity. Our results provide an assay to measure cystatin C activity in CSF and suggest a functional consequence of reduced CSF cystatin C levels in ALS patients.

## Methods

### Samples

For this study, we selected 69 independent CSF samples that were recently utilized in cystatin C biomarker discovery efforts [9]. Sample collection procedures were approved by the institutional review board (IRB) at the University of Pittsburgh, and written informed consent was obtained from all participating subjects. Patient demographics are shown in Table 1. The ALS group included 18 patients with sporadic ALS and five with familial ALS. ALS subjects were diagnosed by experienced neurologists specializing in motor neuron disease, and all were classified as having probable, probable-laboratory-supported, or definite ALS according to El Escorial criteria. Healthy control (HC) research subjects lacked any neurological symptoms, and neurological disease controls (DC) included four patients with multiple sclerosis, two each with primary lateral sclerosis, chronic inflammatory demyelinating polyneuropathy, and progressive muscular atrophy, and one each with spinocerebellar ataxia, small fiber neuropathy, idiopathic sensorimotor polyneuropathy, bilateral facial palsies, neurosarcoidosis, viral encephalitis, CNS lymphoma, brain metastases, pseudotumor cerebri, seizure disorder, complicated migraine, paresthesis with possible myelopathy, and probable conversion disorder.

**Table 1:** Demographic characteristics of study participants

	**ALS (n = 23)**	**Healthy controls (n = 23)**	**Disease controls (n = 23)**
**Sex (male/female)**	11/12	11/12	12/11
**Age at draw ± SD (years)**	48.7 ± 13.9	48.7 ± 14.2	48.5 ± 15.4

There were no significant differences between subject age or gender among groups.

The total cystatin C concentration in each of the 69 CSF samples was previously determined by ELISA [9].

### Immunoprecipitation

For these experiments, the single CSF sample with the highest concentration of cystatin C was used. Immunoprecipitation (IP) of cystatin C from CSF was performed using a magnetic bead system (MACS Molecular, Auburn, CA). First, varying amounts (0, 0.4, 0.8, or 1.6 μg) of affinity-purified goat anti-human cystatin C polyclonal antibody (R&D Systems, Minneapolis, MN) were added to 18 μL aliquots of CSF and the solutions were briefly mixed at room temperature. Next, 100 μL of Protein A MicroBeads were added to each aliquot, and the solutions were mixed and incubated for 30 min on ice. During this incubation, the μ columns (MACS Molecular, Auburn, CA) were each primed with 200 μL of lysis buffer (50 mM Tris HCl, pH = 6.00; 150 mM NaCl; 1% Triton-X-100) and rinsed with 100 μL of assay buffer (see Papain inhibition assay below). Next, the CSF solutions were applied to the μ columns and the "flow-through" was collected. An additional 30 μL of assay buffer were then added to flush the remaining CSF solutions into the same flow-through collection tube. The columns were each rinsed with 50 μL of PBS and the combined flow-through solutions from all three steps were saved. The columns were then rinsed with an additional 750 μL of PBS and 100 μL of low-salt wash buffer (50 mM Tris HCl, pH 6.8; 50 mM DTT; 1% SDS; 0.005% bromophenol blue; 10% glycerol). To elute the isolated cystatin C, 20 μL of pre-heated 1X SDS gel loading buffer were added to each μ column and incubated for 5 min at room temperature. Next, an additional 50 μL of pre-heated 1X SDS gel loading buffer were added and the elution products were collected. For subsequent IPs of flow-through solutions, 4 μL (0.8 μg) of anti-cystatin C antibody and 100 μL of MicroBeads were added to each flow-through sample, and the remaining steps were repeated as above.

### Immunoblotting

IP elution products were separated by electrophoresis on a NuPAGE® Novex® 12% Bis-Tris polyacrylamide gel (Invitrogen, Carlsbad, CA), with 20 μl per gel lane. The proteins were transferred onto a PolyScreen polyvinylidene difluoride membrane (NEN Biolabs, Ipswich, MA) and blocked with 5% nonfat milk in 1X TBS/0.05% Tween-20 (TBST) for one hour at room temperature. A mouse monoclonal anti-human cystatin C primary antibody (Santa Cruz Biotechnology, Santa Cruz, CA) was added to the blocking solution at a 1:1000 dilution and incubated overnight at 4°C. The membrane was thoroughly washed with TBST, and then a goat anti-mouse horseradish peroxidase-conjugated secondary antibody (Millipore, Burlington, MA) was diluted by 1:5000 in 5% nonfat milk in TBST and applied to the membrane for two hours at room temperature. The membrane was thoroughly washed with TBST, and then the labelled proteins were visualized using Chemiluminescence Reagent Plus (PerkinElmer, Waltham, MA).

### Papain inhibition assay

#### Assay procedure

The procedure we developed for this study was modified from an R&D Systems activity assay protocol for recombinant human cystatin C protein (R&D Systems, Minneapolis, MN). Plant-derived papain (Sigma, St. Louis, MO) was diluted to 100 μg/mL in ice-cold activation buffer (50 mM Tris, 5 mM DTT, pH 7) and incubated at room temperature for 15 min. During this incubation, CSF samples from each patient were diluted at a 2:1 ratio by volume in either assay buffer (50 mM Tris, pH 7) or polyclonal cystatin C blocking antibody (R&D Systems, Minneapolis, MN; diluted to 0.2 mg/mL in assay buffer) and incubated with gentle agitation for five min at room temperature. The CSF sample solutions were then further diluted 1:8 in assay buffer, and the activated papain solution was further diluted in assay buffer to 2 ug/mL. Equal volumes of the CSF and papain solutions were mixed and incubated at 37°C for 10 min. We next diluted the mixture 1:5 in assay buffer, and 50 μL were added to each well of a 96-well plate. 50 μL of ZPheArgAMC (R&D Systems, Minneapolis, MN) substrate (10 mM in DMSO, diluted to 200 μM in assay buffer) was rapidly added to each well, and the plate was immediately inserted into a plate reader. The plate was read for 5 min in kinetic mode, using excitation and emission wavelengths of 380 nm and 460 nm, respectively.

#### Assay design

Samples were randomized to plates and plate positions, and assayed in triplicate. Each sample, and its corresponding sample/antibody solution, were always assayed in adjacent wells. A standard curve and an internal standard of mixed CSF were also included on each plate for quality control. The experiment was repeated with samples in reverse order on each plate to control for slight assay differences based on plate position. All experiments were performed at least twice.

### Statistical analysis

All statistics were carried out using SPSS software. Data normality was assessed by the Kolmogorov-Smirnova and Shapiro-Wilk tests, and normality was only assumed when indicated by both tests. All calculated p-values were two-tailed, and the significance level was set at *p* < 0.05.

#### Papain inhibition assay data analysis

For each experiment, the reaction rate for each well was determined as slope of best-fit line of graphed RFU data. Intra-assay coefficients of variation (CVs) were calculated using the three reaction rates for each sample, and each sample/antibody mixture, on each plate. Data from one of the triplicate well readings was dropped from the analyses when it differed from both other wells by a CV ≥ 40, and the other two sample wells differed from each other by a CV less than half of the average CV between the outlier and the similar wells. This eliminated only 0.65% of the assay data points (6 of 930 data points). The average reaction rate for each raw CSF sample (without blocking antibody) was recorded as the total assay activity for that sample. The total assay activity was then subtracted from the average reaction rate for the same sample containing blocking antibody to determine the total cystatin C activity for that sample.

All experiments were performed in triplicate and each experiment repeated at least twice. Inter-assay CVs were also determined for each sample using the total assay activity and, separately, the total cystatin C activity calculated for each separate experiment using the same guidelines described above.

Coefficients of variation between duplicate/triplicate wells for each sample run, and between mean cystatin C activity plate results for each sample, were recalculated after dropping outlier data as described above. Because both groups of CV data failed normality, median values were calculated as the measure of central tendency. The overall intra-assay CV was calculated as the median of the individual CVs for all sample-runs. The CVs were non-normally distributed (range: 0.1–29.9%), and the median (3.6%) was less than the generally acceptable value for intra-assay variability (CV < 10%). Similarly, inter-assay CVs were determined using the final cystatin C activity level determined in each replicate experiment for each CSF sample. The CVs were non-normally distributed (range: 0.2–32.1%), and the median (9.1%) was well within the acceptable range for inter-assay variability (CV < 20%) [15].

#### Correlation analysis

The correlations between cystatin C concentration and both assay activity and cystatin C activity in all samples as a combined group were assessed using non-parametric statistics because the data for total assay activity were non-normally distributed. For these analyses, SPSS software was used to calculate both the Spearman correlation coefficient and the two-tailed significance level. Identical methods were used to assess the same correlations in each diagnostic group because cystatin C concentrations in ALS patients also failed normality testing.

#### Comparison between diagnostic groups

Because cystatin C activity ratios were normally distributed in all three experimental groups and there were no significant differences among group variances, we compared group means by one-way ANOVA.

For our final comparison of cystatin C concentration, total assay activity, and total cystatin C activity between groups, all diagnostic group data were normally distributed except for cystatin C concentration. For each data category, differences among diagnostic group means were identified by one-way ANOVA.

#### Analysis of samples paired by cystatin C concentration

Samples in all three diagnostic groups were paired by cystatin C concentration, and the seven samples in each group which could not be paired with samples from the other groups were dropped from this analysis. All paired data for cystatin C concentration, total assay activity, total cystatin C activity, and cystatin C activity ratio were normally distributed. Therefore, we were able to use the SPSS general linear model for repeated measures to assess differences in each of the four variables among diagnostic groups.

## Results

### Papain inhibition assay to measure cystatin C activity

To measure cystatin C protease inhibitory activity in the cerebrospinal fluid (CSF), we utilized a papain inhibition assay. Papain is a plant-derived cysteine protease that can hydrolyze the Arg-AMC amide bond of a Z-Phe-Arg-AMC substrate to release the fluorescent 7-amino-4-methyl coumarin (AMC) group. Relative fluorescence units (RFU) are then measured over time to determine the total papain activity. When a cysteine protease inhibitor (CPI) is added to the assay solution, it inhibits papain-mediated Z-F-R-AMC substrate cleavage, and fluorescence emission is reduced. The total activity of the CPI can then be estimated as the reduction in papain-mediated assay activity.

There are several challenges when using this assay to measure the activity of a specific CPI within a biofluid sample, such as CSF. First, because CSF contains several CPIs, the total assay inhibition is a measure of the combined CPI activity and cannot be attributed to a single protein. Additionally, CSF also contains endogenous cysteine proteases that could potentially cleave the Z-F-R-AMC substrate and supplement papain activity. This would increase the overall fluorescence emission and decrease the apparent CPI activity determined by the assay.

To control for these factors, we determined the total cystatin C activity in CSF by assaying both raw CSF, and CSF from the same patient sample that had been pre-incubated with an antibody that blocks cystatin C activity. For raw CSF samples, the total measured assay activity included the supplementary effects of all endogenous cysteine proteases as well as the inhibitory effects of all CPIs present in the sample. For the CSF treated with the blocking antibody, the measured assay activity included all of the factors present in raw CSF except for the inhibitory activity of cystatin C. Therefore, the total inhibitory activity of cystatin C in each sample could be calculated by subtracting the total assay activity for raw CSF from the total assay activity for CSF treated with the blocking antibody.

### Specificity and efficiency of cystatin C blocking antibody

**Figure 1:**
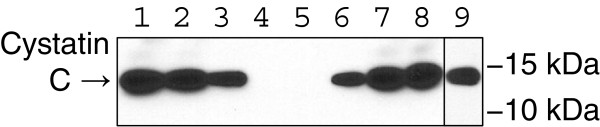
**Depletion of cystatin C from CSF by immunoprecipitation.** Lanes 1–4: Increasing amounts of anti-cystatin C polyclonal antibody (0–1.6 μg antibody) was added to CSF for immunoprecipitation as described in Methods. The resulting immunoprecipitate was analysed by western blot for cystatin C. Lane 1: 1.6 μg antibody (IP1); Lane 2: 0.8 μg antibody (IP2); Lane 3: 0.4 μg antibody; (IP3) Lane 4: 0 μg antibody (IP4). The remaining column flow-through from each sample was then used to repeat the cystatin C immunoprecipitation using 0.8 μg antibody to examine efficiency of cystatin C removal during the initial immunoprecipitation. Lane 5: Flow-through from IP1; Lane 6: Flow-through from IP2; Lane 7: Flow-through from IP3; Lane 8: Flow-through from IP4; Lane 9: Human cystatin C purified protein as a positive control from a separate gel.

Experiments were first conducted to test the effectiveness of the polyclonal anti-cystatin C antibody in both binding to human cystatin C within CSF and in eliminating cystatin C activity from CSF samples. First, varying amounts of the antibody were used to immunoprecipitate (IP) cystatin C from the CSF. Western blots were then performed to assess the relative amounts of cystatin C removed from each sample aliquot by IP, as well as the amount remaining in each sample (Figure 1). We found that the selected antibody effectively precipitated cystatin C from human CSF, and that 1.6 μg of antibody (in 8 μL volume) was adequate to fully remove cystatin C from 18 μL of CSF (approximate 2:1 CSF to antibody volumetric ratio).

Next, CSF aliquots from the same sample were pre-incubated with varying amounts of the same polyclonal "blocking" antibody and used in the papain inhibition assay as described in Methods. We observed an antibody dose-dependent elimination of cystatin C inhibitory activity, with full elimination of function achieved by a sample to antibody ratio of 2:1, by volume in a CSF sample that contained the highest amount of cystatin C protein as determined by ELISA (Table 2). This antibody ratio was effective at fully eliminating activity in numerous CSF samples across a range of cystatin C protein levels (data not shown). These findings confirm that this polyclonal antibody can effectively bind to human cystatin C and eliminate its functional activity from human CSF samples.

**Table 2:** Dose-dependent effect of cystatin C activity-blocking antibody

**Sample**	**CSF to antibody ratio (by volume)**	**Mean assay activity (RFU/min)**
**1**	0:4	3737
**2**	4:0	754
**3**	4:1	2455
**4**	4:2	3944
**5**	4:3	3799
**6**	4:4	3603

A CSF sample with the highest cystatin C concentration was pre-incubated with varying amounts of polyclonal cystatin C activity-blocking antibody (samples 2–6) and then tested in the papain inhibition assay. Sample 1 demonstrates the baseline papain assay activity in the absence of CSF-mediated papain inhibition and in the presence of the highest dose of anti-cystatin C antibody used in the experiment. Sample 2 demonstrates the maximal papain assay inhibition produced by the CSF without any anti-cystatin C antibody. Addition of the blocking antibody in samples 3–6 produces a dose-dependent elimination of CSF-mediated assay inhibition, with full elimination reached at a 4:2 ratio (where assay activity first returns to baseline). Cystatin C antibody was prepared at a 0.2 μg/μL concentration.

### Relationship between CSF cystatin C concentration and activity

**Figure 2:**
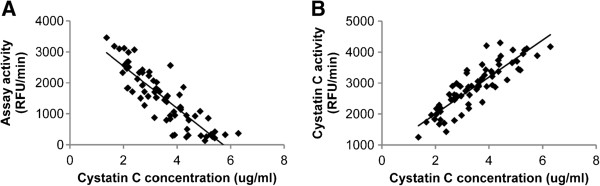
**Correlation of cystatin C concentration with papain inhibition assay activity and cystatin C activity.** (**A**) Total assay activity was indirectly related to cystatin C concentration, and the relationship was statistically significant (p < 0.001, Spearman r = -0.861). (**B**) Total cystatin C activity was directly related to its own concentration, and the correlation was strong and significant (p < 0.001, Spearman r = 0.883).

This 2:1 sample to antibody ratio was used to perform the papain inhibition assay in all CSF samples to determine cystatin C functional activity in each sample. Before separating the data into diagnostic groups, we graphed both total assay activity and total cystatin C activity versus the cystatin C concentration for each sample. We observed a strong, indirect relationship between CSF cystatin C concentration and total papain assay activity (Figure 2A), and a strong, direct relationship between cystatin C concentration and its own activity (Figure 2B). Next, the data were sorted into diagnostic categories and these correlation analyses were repeated. The relationships observed in the combined data were maintained in all three individual diagnostic categories, and all correlations were statistically significant, with *p* < 0.001 and Spearman r values between -0.86 to -0.91 for each individual subject group cystatin C and total papain assay activity.

### Comparison between diagnostic groups

We next compared CSF cystatin C concentration and activity levels among the three diagnostic groups (Table 3). The mean cystatin C concentration was reduced in ALS patients compared to the DC and HC groups as previously reported [9], but this difference did not reach statistical significance. The low mean cystatin C concentration in ALS patients corresponded with the greatest mean papain assay activity out of the three diagnostic groups. The differences in mean cystatin C activity between diagnostic groups directly mirrored the group trends in mean cystatin C concentration and did not reach statistical significance (Table 3). Cystatin C activity ratios were calculated for all samples by dividing the total cystatin C activity by the total cystatin C concentration for each sample. Our results suggest that the average protease inhibitory activity per unit of cystatin C was equivalent across all groups (Table 3).

**Table 3:** Comparison of cystatin C concentration, papain assay activity, cystatin C activity, and cystatin C activity ratio among experimental groups

	**Mean cystatin C concentration ± SD**	**Mean assay activity ± SD**	**Mean cystatin C activity ± SD**	**Mean activity ratio ± SD**
**ALS (n = 23)**	3.09 ± 1.10	1744 ± 919	2663 ± 762	887 ± 131
**DC (n = 23)**	3.51 ± 1.07	1547 ± 980	2868 ± 850	829 ± 133
**HC (n = 23)**	3.84 ± 1.13	1302 ± 742	3124 ± 634	843 ± 138
***p*-value**	0.077	0.247	0.124	0.312

For each diagnostic group, the mean cystatin C protein concentration in the CSF (μg/ml), papain inhibitory assay activity in raw CSF, and cystatin C activity in CSF treated with anti-cystatin C antibody are shown. The right column depicts the mean activity ratio determined by dividing the total cystatin C activity by the total cystatin C concentration for each sample. No differences among experimental groups reached statistical significance by one-way ANOVA. Assay activity units are listed in RFU/min.

Finally, to fully control for differences in mean cystatin C concentration among diagnostic groups, we paired select data from each group for statistical comparison. Subsets of samples with matched cystatin C concentrations were selected from each group, generating three diagnostic subgroups with identical mean cystatin C protein concentrations (Table 4). We then compared activity ratios using statistics for repeated measures in order to identify any differences in the kinetics of cystatin C activity among groups. In this analysis, no significant differences were observed among the three diagnostic groups, and all trends were eliminated by controlling for cystatin C protein concentration (Table 4).

**Table 4:** Repeated measures assessment of cystatin C activity

	**Mean cystatin C concentration (μg/ml) ± SD**	**Mean assay activity ± SD**	**Mean cystatin C activity ± SD**	**Mean activity ratio ± SD**
**ALS (n = 16)**	3.54 ± 1.03	1381 ± 798	3006 ± 652	875 ± 132
**DC (n = 16)**	3.54 ± 1.05	1476 ± 1009	2939 ± 827	842 ± 149
**HC (n = 16)**	3.53 ± 1.03	1433 ± 782	3010 ± 691	875 ± 121
***p*-value**	0.597	0.796	0.843	0.859

When samples were paired across groups by CSF cystatin C concentration, there were no significant differences in papain assay activity, cystatin C activity, or cystatin C activity ratio among the three diagnostic groups. Assay activity units are listed as RFU/min.

## Discussion

Prior studies have reported that cystatin C concentrations are decreased in the CSF of ALS patients relative to healthy controls, but it has been unclear if these reductions in protein level result in proportional reductions in cysteine protease inhibitory activity. To address this important question, we modified a papain inhibition assay to allow for the direct measurement cystatin C functional activity in human CSF. The assay performed with low inter- and intra-assay variability, and allowed the reliable evaluation of cystatin C activity in multiple CSF samples from ALS patients and controls. Notably, this assay measures only the activity of cystatin C at its primary active site, which interacts with papain-like cysteine proteases. Therefore, the experimental findings of this study only apply to the activity of cystatin C against its primary ligands, and do not address its interactions with legumain-like cysteine proteases.

As expected, cystatin C concentration demonstrated a strong, direct relationship with the magnitude of its inhibitory activity (Figure 2). This confirms that CSF cystatin C is biologically active against papain-like cysteine proteases, and that there does not appear to be significant variability in the activity state of the protein among individual subjects.

Cystatin C concentration also demonstrated a strong, indirect relationship with the overall activity of the papain assay (Figure 2). This indicates that CSF induces an overall inhibitory effect on papain-mediated protease activity, and that this effect is highly dependent upon cystatin C concentration. Additionally, the strength of the correlations between cystatin C concentration and both its own activity (Figure 2B) and total papain assay activity (Figure 2A) were nearly identical in magnitude (r of 0.883 and -0.861, respectively). This, along with Table 2 data indicating that cystatin C antibody can completely block papain inhibition in CSF, suggests that any other endogenous papain-like cysteine proteases and alternate cysteine protease inhibitors that are present in human CSF contribute only minimally to the measured assay activity. Therefore, cystatin C is likely the dominant cysteine protease inhibitor in human CSF, and plays a critical role in the regulation of cysteine protease-mediated proteolysis in this setting. While prior studies have shown that cystatin C protein levels are enriched in the CSF versus the blood and the protein is often detected in proteomic studies of the CSF [16,17], our data suggests it is the primary cysteine protease inhibitor in the CSF. It should be noted that if cystatin C concentrations approach saturating concentrations for their selected cysteine proteases in the CSF, then small changes in concentration between individuals or subject groups may not translate to significant physiological changes in inhibitory activity.

When the data were divided into diagnostic groups, comparable correlations were maintained between cystatin C concentration and both its own activity and total papain assay activity in each group (Table 3). Activity ratios were then calculated for each patient as the total cystatin C activity divided by the total cystatin C concentration, and the resulting mean values were nearly identical between the three experimental groups (Tables 3 and 4). However the large intragroup variations suggest that cystatin C may not be useful as an individual biomarker for ALS but could be useful in a panel of disease biomarkers.

Reduced CPI activity within the CSF of ALS or other neurologic disease patients may contribute to either toxic or protective pathways related to disease pathogenesis. Cystatin C has been reported to exhibit neurotoxic effects when injected into the rodent brain [18], and when directly applied to primary neuronal cultures [19]. However, cystatin C also exhibits neuroprotective effects both *in vivo*[20] and *in vitro*[21], and potential neuroprotective pathways of cystatin C have been more thoroughly characterized [22]. The protective function that appears most relevant to ALS is the regulation of extracellular cathepsins and calpains, which are released as part of physiological processes or in response to CNS damage or stress. The levels of these proteases may be elevated in ALS and other diseases due to increased expression [23,24], secretion by activated microglia [25], and release by dying neurons [24,26-29]. Therefore, the reduced CSF cystatin C activity in ALS may be inadequate to counteract the apparent increase in protease activity, resulting in protease-mediated CNS damage.

Extracellular cystatin C also may be internalized by motor neurons and/or glial cells, and subsequently affect intracellular processes. Efficient cystatin C uptake has been demonstrated with multiple non-neuronal human cell lines [30]. If this process also occurs in the human CNS, reduced extracellular cystatin C concentration could result in reduced uptake and abnormal deficiencies in intracellular cystatin C activity. Intracellular cathepsins and calpains both appear to be up-regulated in ALS, and can contribute to the induction of apoptosis [23,24]. Therefore, deficiencies in intracellular cystatin C could potentially lead to apoptosis through the loss of a protective mechanism. Alternatively, reduced extracellular cystatin C levels may reflect increased cellular uptake in response to increased intracellular levels of cathepsins and calpains.

## Conclusions

We have determined that the functional activity of cystatin C within the CSF can be effectively measured by a papain inhibition assay, and that CSF cystatin C activity is directly proportional to its concentration in ALS patients and controls. Our data indicates that cystatin C is the predominant cysteine protease inhibitor in the human CSF.

## Competing interests

Robert Bowser is co-Founder of Knopp Biosciences LLC and Iron Horse Diagnostics Inc., biotechnology companies devoted to the development of diagnostics and therapies for ALS and other neurological diseases.

## Authors’ contributions

Conceived and designed the experiments: MW and RB. Performed the experiments: IB and MW. Analyzed the data: MW. Wrote and revised the paper: MW and RB. Edited and revised the paper: IB. All authors read and approved the final manuscript.
